# Editorial: Early detection and combination therapy for diabetic peripheral neuropathy: what does the future look like?

**DOI:** 10.3389/fendo.2024.1422734

**Published:** 2024-05-29

**Authors:** Mark Yorek

**Affiliations:** ^1^Department of Internal Medicine, University of Iowa, Iowa City, IA, United States; ^2^Department of Veterans Affairs Iowa City Health Care System, Iowa City, IA, United States; ^3^Fraternal Order of Eagles Diabetes Research Center, University of Iowa, Iowa City, IA, United States

**Keywords:** diabetes, diabetic neuropathy, prediabetes, peripheral neuropathy, treatment

The goal of this Research Topic was to highlight the importance of early detection of nerve damage and early repair in diabetic peripheral neuropathy (DPN). For many diseases, early detection is important for successful treatment. For DPN there is no recommended treatment other than good glycemic control, and this is not overly successful in patients with type 2 diabetes. This lack of a satisfactory treatment can be attributed to many factors including a poor understanding of etiology, the lack of effort to pursue combination therapies, and the inability to diagnose DPN in its early stages. There are no screening recommendations to identify patients with prediabetes due to its early stage. Three articles in this Research Topic will address means of early detection of peripheral neuropathy and whether evidence exists of neuropathy in patients at high risk for prediabetes. Another article will revisit pre-clinical studies of diabetic rodents to determine what combination therapies could have the potential for best outcome for DPN management.

Kababie-Ameo et al. (*Evidence of impaired H-reflex and H-reflex rate-dependent depression in diabetes, prediabetes, and obesity: a mini-review*) in their review article discussed the inadequacy of current screening tests for peripheral neuropathy not only in patients with diabetes but also in those with prediabetes or obesity. These tests include tendon reflex, temperature sensation, and pressure and vibration perception, which are subjective and require a high degree of evaluator experience. As an alternative, they reviewed the literature on the use of an electrophysiology test known as the Hoffmann reflex (H-reflex). This test evaluates the excitability of the α-motoneurons where a prolonged latency is an indicator of neuronal damage. The authors concluded that, when accompanied by other clinical tests such as the Michigan Neuropathy Screening Instrument, the absence or prolonged latency of the H-reflex is significantly associated with a predictive value for DPN.

The article by Liu et al. (*Development and validation of a risk prediction model for early diabetic peripheral neuropathy based on a systematic review and meta-analysis*) examined a model that combined 11 common clinical indicators for early prevention and intervention of DPN in patients with type 2 diabetes. The authors found that by combining age, smoking, BMI, duration of diabetes, HbA1c, low HDL-c, high triglycerides, diabetic retinopathy, and kidney and cardiovascular disease along with lifestyle provided a reliable risk prediction model for DPN.

Körei et al. (*No clear evidence of neuropathy among patients with high risk for the development of prediabetes/diabetes – a pilot study*) conducted a study looking at the risks of developing prediabetes/diabetes and autonomic or sensory neuropathy. Their results suggest that individuals at an increased risk of developing prediabetes/diabetes are not at a higher risk of developing autonomic or sensory neuropathy during the course of disease. This work emphasizes the difficulty physicians and care givers face in trying to identify patients that may benefit most from early intervention in order to delay development and progression of peripheral neuropathy.

Yorek (*Combination therapy is it in the future for successfully treating peripheral diabetic neuropathy*)*?* presented a review article that questioned the logic of pursuing a monotherapy for the treatment of DPN. In this article, the complexity of the etiology of DPN, which included the multiple pathophysiological pathways, was discussed. The author discussed the different treatments for DPN that have been used in preclinical studies with success. However, these treatments failed to provide relief of disease when applied to human patients. The reasons for the failure of translating successful treatments for DPN in animal models to humans has been hotly debated for many years. In this article, the author places partial blame on the unrealistic view that a mono-therapeutic approach exists. In the article an analogy between treatment of hypertension and DPN is made. Combination therapy is widely used to normalize blood pressure. Hypertension, like DPN, is a disease with multiple etiologies and there is no hesitation to use multiple drugs, with dosage often being adjusted to obtain the desired result. Therefore, the author states that it seems logical that combination therapy combined with lifestyle adjustments will be required to successfully treat DPN.

In summary, this Research Topic attempted to highlight challenges faced by researchers and physicians in seeking a treatment for DPN. Over the years there has been a lot of work done by talented people, but more work is needed before we obtain success.

## Author contributions

MY: Writing – review & editing, Writing – original draft.

